# Left-handed cardiac surgery: tips from set up to closure for trainees and their trainers

**DOI:** 10.1186/s13019-016-0523-y

**Published:** 2016-09-01

**Authors:** Clare Burdett, Joel Dunning, Andrew Goodwin, Maureen Theakston, Simon Kendall

**Affiliations:** Department of Cardiothoracic Surgery, James Cook University Hospital, Marton Road, Middlesbrough, TS4 3BW UK

**Keywords:** Left-handed, Lefthanded, Left handed, Cardiothoracic, Surgery, Surgical training

## Abstract

There are certain obstacles which left-handed surgeons can face when training but these are not necessary and often perpetuated by a lack of knowledge. Most have been encountered and overcome at some point but unless recorded and disseminated they will have to be resolved repeatedly by each trainee and their trainers. This article highlights difficulties that the left-hander may encounter in cardiac surgery and gives practical operative advice for both trainees and their trainers to help overcome them.

## Background

The material components of our world are designed for right-handers [1]. This is unsurprising as they outnumber left-handers 9 to 1 [2]. All objects or processes that express a lateral bias are potential obstacles to the left-handed surgical trainee. Nonetheless left-handers can and do make good cardiac surgeons. There are even aspects of cardiac surgery that favour the left-hander: such as the orientation of the left ventricular outflow tract for valve and root replacement. Through necessity left-handers are generally practised adaptors to the right-handed world. However there is a limit to the degree of compromise. The precision and consistency demanded in cardiac surgery makes provision of the correct tools essential for left-handers to match the performance of their right-handed colleagues. Left-handed surgical instruments do exist for this purpose.

With forethought and some limited modifications to practice left-handed surgeons can effectively work alongside and be trained by right-handers. So why do so many encounter difficulties during their training? Unfortunately, surgical traditions and misconceptions continue to hamper left-handed training [3]. Lack of information, or rather its dissemination hinders change. Owing to the disproportionate numbers, many left-handed trainees are isolated from left-handed surgeons. Most right-handed surgeons (all of whom could be good potential trainers) will have encountered only a few left-handed trainees. As a result, both trainers and trainees can end up struggling to ‘re-invent the wheel’ in overcoming difficulties encountered and solved already by others.

By providing information on best practice these ingrained barriers can be broken down to reveal how straightforward training a left-handed cardiac surgeon should be.

## Preparation

### Use your left hand

#### Unless you are truly ambidextrous, do not try to become right-handed

This is not always obvious. Trainees receive mixed advice on whether to switch, even on official surgical skills courses. Life is easier as a right-hander; equipment is plentiful and you can directly copy your trainer’s moves. However, unless you are truly ambidextrous you automatically put yourself at a disadvantage. Surgical training is competitive and opportunities to impress are finite. Self-evidently the left-hander masquerading as a right-hander does not have the same ‘dexterity’ as his or her right-handed counterpart. The training paradox is that looking slick initially, rather than an evident need for more practice, is what gets you cases. For those further down the line, changing back and re-training with you left-hand is possible. There are trainees who have successfully re-learnt and benefited technically from doing so, but this still requires perseverance and a supportive environment from trainers.

### Use left-handed instruments

#### Essential

The tools exist but access to them is limited. Why? Instruments are costly but fundamental to surgical practice. Right-handed equipment is purchased with minimal hesitation. However, there is a lack of appreciation for the value of left-handed sets in training. As such, they are viewed as an optional and unwelcome expense.

We have written a separate article on instrumentation [4] which explains why and what is needed. Most left-handed trainees have struggled to get access to equipment. Trainees are in a weak position when negotiating for left-handed instruments; they are not permanent members of staff and hold a junior position. It can be difficult to convince seniors of their value and even willing trainers are unsure how to make a compelling case for raising the funds. Ironically it may have been easier in the past to obtain left-handed equipment when senior surgeons had more control over departmental budgets.

Some trainees have attempted their own solutions. Purchasing your own equipment is possible but affordability limits quality and you can no longer transfer sets between hospitals. At least one trainee has secured funds from an industrial sponsor, but this is only an option in the latter years of training. Locating yourself in a hospital with a left-handed consultant is a pragmatic solution for the individual. On a national scale it would simply cause clustering of left-handed trainees and is unlikely to be sustainable for the entirety of an individual’s training.

This barrier has proved very difficult to overcome. Hopefully, the growing acknowledgement of the importance of left-handed equipment in training will help trainees negotiate more effectively at deanery or hospital level. Trainers need to be supportive for this to succeed.

### Engage the wider team

#### Communication is required to ensure co-operation in theatre

As a left-hander in an operating department it is hard to know what adaptions you can realistically expect others to make. All the suggestions below in the section ‘intra-operative tips’ are reasonable requests and have been used by left-handers elsewhere. The left hander can expect some resistance when breaking the ‘routine’ and this can be eased by skilled negotiation with all those concerned such as the trainer, scrub nurse, and anaethetist. Clearly strong support from the trainer is pivotal.

## Intra-operative tips

Like right-handers, no two left-handers operate the same way. However, in researching for this article we have spoken to left-handed trainees and their left and right-handed consultant trainers. We have documented the common themes which emerge. The following are tips for the generic parts of a cardiac operation through a median sternotomy.

### Set-up

#### Equipment

Check with the scrub nurse in good time that he/she has your left-handed instruments. Indeed this should be highlighted the day before surgery. If the scrub nurse is not used to loading needles for a left-hander – help show them how (see the article on instruments). Although not difficult it can cause confusion and requires re-mounting of the needle. Repeated remounting makes a significant difference to the flow and speed of the operation, and it can also become a source of irritation for the trainer.

#### Antiseptic preparation and draping

This is really important - ensure that the anaesthetic team place the bar far back above the patient’s head beyond the cranium as left-handers require elbow-room to operate effectively. This does not prevent anaesthetic observation of the patient and is an adjustment that is routinely made in many centres. Right-handers do not have this problem because their dominant arm can move freely above the thorax and abdomen.

#### Positioning

Occasionally there is the suggestion that a left-hander should ‘swap sides’ and operate from the patient’s left. This is non-sensical as the heart naturally lies towards the left under the left sternum – and therefore access is more difficult standing on the patient’s left side.

### Opening

#### Knife to skin

It is easier for a left-hander to incise the skin from xiphisternum to sternal notch. The angle at which you can stand is better. This may challenge some consultants’ ideals on mid-line incisions, however it can be done. If you make your incision slightly above the notch you will have more room to manoeuvre your needle holder when creating your purse strings for cannulation.

#### Sternal incision

If you use your dominant hand to hold the saw then you start at the notch. If you prefer to guide with you left hand then you will start at the xiphisternum.

### Cannulating

There are many different ways to cannulate the aorta – one or two purse strings, snugged at the base or to the sides. The angles and number of fore and back-hand shots will be different for you and your right-handed trainer, which may make an easy shot for them difficult for you. Practise at home and adapt the sequence if this is the case. The same applies for the venous pipe.

If you incise the aorta with your left hand (which gives you most control) you can hold the cannula with your right hand below the incision and then insert it. The important point is to not cross your hands, which may happen if you try to copy a right-hander’s moves.

### Conduit

#### Leg vein

Often this is the first part of an operation a trainee will be allowed to do. However, the novice left-hander can encounter problems. For instance, vein is often taken from the ankle working up towards the knee. If the operator stands on the right side of the table lateral to the leg and uses scissors to cut the skin, the right-hander has a direct forward run. The left-hander has to contort their arm to an un-natural position to cut.

Alternative strategies for overcoming this are: -

- Stand on the other side of the table (this will displace the scrub nurse when they are set up on the patient’s left)
- Stand at the end of the bed (you have to stretch to get more than one length of vein)
- Start at the groin and work down with scissors (the vein is deeper here so learn how to locate it)
- Start at the ankle and use a scalpel (this utilises a pulling action, which is a more natural movement in this context)

#### Mammary artery

There is little consensus on where to start – both cranial and caudal are accepted techniques. Your hand will not obscure your view if you correctly position yourself to look up towards the artery. Then it does not matter whether you work towards or away form yourself. Accordingly, the myth that the right mammary artery is easier for the left-hander is unfounded.

### AVR and CABG

Ergonomically, the left-hander is well placed to undertake aortic surgery as their dominant hand lies above the aortotomy incision (when looking down towards the valve orifice). Going clockwise or anti-clockwise is discretionary. To ensure accurate suture placement, the aortotomy closure is started from the right side (non-coronary sinus). It can be accomplished forehand by pointing the elbow towards the assistant and suturing the proximal aortic edge, followed by the distal. Backhand allows the distal aortic edge to be sutured first. Coronary anastomoses (both proximal and distal) are performed in many different ways (amongst right-handed as well as left-handed surgeons), and there is no correct way. When training, it is useful to try different techniques, to ultimately establish your own. The important point, is that right-handed trainers need to be patient, as an easy shot for them may be a difficult one for the left-handed trainee initially. Equally, there will be some moves that are easier. For instance, the right top end is easier for lefthanders as the vein graft is held to the right of the aortic punch hole. Often a right-hander’s forehand will be a left-handers backhand, and as such using a technique developed specifically by a left-handed surgeon may contain more forehand passes. You can refer to our videos on-line which show a left-handed surgeon performing these procedures, demonstrating a reproducible sequence that is efficient for the left-hander [5].

### Coming off bypass

The easier task is removing the pipe, so use you right hand and leave your left hand free to gain control – by either covering the hole or snugging. Your right is also the hand closest to the scrub nurse which facilitates an easy transfer of the pipe.

### Closure

#### Sternum

You can close the sternum left-handed with a left-handed wire holder. You may need to place your back towards the patient’s head to get the right angle for your first shot through the manubrium.

#### Soft tissues

A left and right-hander standing opposite one another are both likely to want to start at the same end of the incision. Practise both so that you can adapt as necessary. But where possible the ‘operating’ surgeon should start caudally and close the linea alba and as a left-hander you can get a good view of the tissues to be sutured at this ‘important’ end of the wound.

## Other scenarios

### Emergencies

You can use the generic kit supplied in an emergency re-opening. Scalpel, wire cutters and retractor work equally well for the left and right-hander. For removing wires, the right-handed needle holder is adequate. If the situation allows there might be time for your left-handed equipment to be fetched, but not at the expense of patient safety.

### Assisting

Most equipment in the operation will be generic and therefore right-handed (for example artery clips and cross-clamps). Using them does not require the same level of skill as suturing. Learn how to open them with your right and left hand. All trainees should be capable of assisting with both hands, so you are not at a disadvantage.

Left-handed instruments may be scarce. If so, it is reasonable to keep some items unopened but ensure they are in theatre close at hand just incase a spontaneous learning opportunity arises.

## Trainers

### The right-handed trainer

#### The majority of your trainers will be right-handed and this can be a good thing

Throughout your career, you will work with more right-handers than left. Learning how to assist them, how to be assisted by them and how to train future right-handers are all important skills to master.

Whilst some right-handed trainers are agnostic about training left-handers, most left-handed trainees have been hampered by bias at some point during their training. Some bosses claim they cannot train left-handers, others have more specific complaints – such as the dizzy feeling induced in one by watching a left-hander go round an anastomosis ‘the wrong way’!

Actually some of the best trainers of left-handers have been right-handed. Willingness to try is actually the most important factor. Accepting that routines may have to be changed or adapted is also important. Trainer and trainee can work together to overcome technical difficulties, and the growing number of publications and web-based tutorials on left-handed techniques should help speed this process up [5, 6].

### The left-handed trainer

#### If you get this opportunity – take it!

Aiming to work solely with left-handers is not advisable or indeed practical. However, there are certain points during your training where it can be immensely helpful, especially when you are starting and need advice on instruments and basic techniques. It is also useful later on to perfect certain procedures or overcome specific difficulties that you are having.

If you cannot organise a rotation with a left-hander, visiting one to observe and discuss technical aspects can also be beneficial. There are now tutorials by left-handed consultants available on YouTube via the Left-Handed Cardiac Surgeons Club [5]. When working for a right-hander, you and your boss can both speak to left-handed trainees or consultants for informal advice.

## Conclusions

Barriers to the progress of the left-handed trainee clearly do exist. At times they can seem insurmountable, especially when working in isolation from other left-handers. However, they can be removed with the co-operation of other parties. Your trainer can be right or left-handed but their support and understanding is a paramount factor in your success. Providing them with information on proven left-handed training practices used elsewhere may enhance training opportunities and thereby accelerate your progress.

## Abbreviations

AVR, aortic valve replacement; CABG, coronary artery bypass grafting
